# Bis[5-(2-amino-3-pyrid­yl)tetra­zolato]copper(II)

**DOI:** 10.1107/S1600536809034369

**Published:** 2009-09-05

**Authors:** Min Guo

**Affiliations:** a Ordered Matter Science Research Center, Southeast University, Nanjing 210096, People’s Republic of China

## Abstract

In the centrosymmetric title complex, [Cu(C_6_H_5_N_6_)_2_], the Cu^II^ ion is coordinated by four N atoms from two symmetry-related bidentate 5-(2-amino-3-pyrid­yl)tetra­zolate ligands in a slightly distorted square-planar environment. There are weak intra­molecular N—H⋯N hydrogen bonds between the two ligands. In the crystal structure, there are significant π–π stacking inter­actions between symmetry-related tetra­zole and pyridine rings, with a centroid–centroid distance of 3.6025 (18)°.

## Related literature

For the coordination chemistry of tetra­zole compounds, see: Butler (1984 ▶); Zhao *et al.* (2008 ▶). For the *in situ* [2 + 3] cyclo­addition synthesis of tetra­zole coordination polymers, see: Xiong *et al.* (2002 ▶); Ye *et al.* (2006 ▶); Fu *et al.* (2008 ▶). For coordination polymers synthesized with similar organic ligand derivatives, see: Ye *et al.* (2005 ▶); Bhandari *et al.* (2000 ▶).
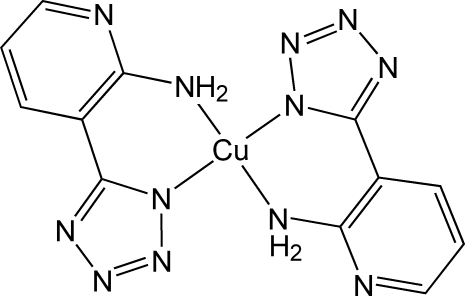


## Experimental

### 

#### Crystal data

[Cu(C_6_H_5_N_6_)_2_]
*M* *_r_* = 385.86
Monoclinic, 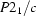

*a* = 6.6492 (6) Å
*b* = 7.9093 (7) Å
*c* = 13.5581 (12) Å
β = 100.692 (2)°
*V* = 700.65 (11) Å^3^
*Z* = 2
Mo *K*α radiation
μ = 1.59 mm^−1^
*T* = 294 K
0.15 × 0.13 × 0.09 mm

#### Data collection

Rigaku Mercury2 diffractometer
Absorption correction: multi-scan (*CrystalClear*; Rigaku, 2005 ▶) *T*_min_ = 0.797, *T*_max_ = 0.870
3743 measured reflections
1363 independent reflections
1228 reflections with *I* > 2σ(*I*)
*R*_int_ = 0.020

#### Refinement

*R*[*F* ^2^ > 2σ(*F* ^2^)] = 0.040
*wR*(*F* ^2^) = 0.109
*S* = 1.07
1363 reflections
115 parameters
H-atom parameters constrained
Δρ_max_ = 0.69 e Å^−3^
Δρ_min_ = −0.73 e Å^−3^

### 

Data collection: *CrystalClear* (Rigaku, 2005 ▶); cell refinement: *CrystalClear*; data reduction: *CrystalClear*; program(s) used to solve structure: *SHELXS97* (Sheldrick, 2008 ▶); program(s) used to refine structure: *SHELXL97* (Sheldrick, 2008 ▶); molecular graphics: *PLATON* (Spek, 2009 ▶); software used to prepare material for publication: *SHELXTL* (Sheldrick, 2008 ▶).

## Supplementary Material

Crystal structure: contains datablocks I, global. DOI: 10.1107/S1600536809034369/lh2827sup1.cif

Structure factors: contains datablocks I. DOI: 10.1107/S1600536809034369/lh2827Isup2.hkl

Additional supplementary materials:  crystallographic information; 3D view; checkCIF report

## Figures and Tables

**Table 1:** Selected bond angles (°)

N5^i^—Cu1—N5	180
N5—Cu1—N4	89.03 (10)
N5—Cu1—N4^i^	90.97 (10)
N4—Cu1—N4^i^	180

Symmetry code: (i) 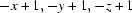.

**Table 2:** Hydrogen-bond geometry (Å, °)

*D*—H⋯*A*	*D*—H	H⋯*A*	*D*⋯*A*	*D*—H⋯*A*
N5—H5*A*⋯N2^i^	0.90	2.50	2.902 (4)	108

Symmetry code: (i) 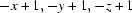.

**Fig. 1.**
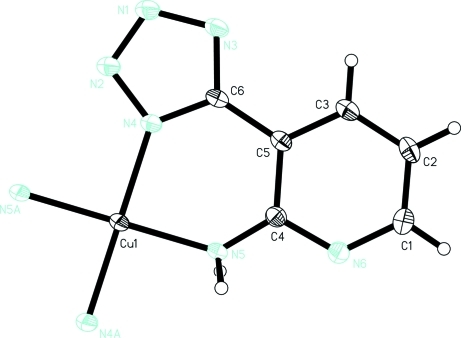
The molecular structure of the title compound with the atomic numbering scheme. Displacement ellipsoids were drawn at the 30% probability level. Symmetry code: (a) -x+1, -y+1, -z+1. Hydrogen bonds are shown as dashed lines.

## supplementary crystallographic information

### Comment

In situ [2+3] cycloaddition synthesis of tetrazole coordination polymers under hydrothermal conditions has proved to be a fast and convenient route to explore novel coordination polymers with rich structural diversities and potential physical properties, such as second harmonic generation (SGH), ferroelectric and dielectric responses (Xiong *et al.*,  2002; Ye *et al.*,  2006; Fu *et al.* (2008). The crystal structure of the compound formed by our hydrothermal synthesis is reported herein.

The molecular structure of the title compound is shown in Fig. 1. The Cu^II^ ion  lies on an inversion center coordinated by four N atoms from two symmetry related bidentate 3-(2-Amino-pyridyl))tetrazolato ligands in a slightly  distorted square-planar environment. In the crystal structure, there are significant π–π stacking interactions between symmetry related tetrazole and pyridine rings with a centroid to centroid distance of 3.6025 (18)°.

### Experimental

The title compound was prepared by hydrothermal treatment of 2-aminonicotinonitrile (2.3 mmol) and Cu(NO_3_)_2_ (1.0 mmol) with excess amount of NaN_3_ in a sealed Pyrex tuble at 403K for 2–4 days. The resulting green rectangular crystals gave a yield of 75% based on Cu(NO_3_)_2_.

### Refinement

H atoms were placed in caculated positions with C-H = 0.93 and N-H = 0.90Å and refined using a riding-model approximation with U_iso_(H) = 1.2U_eq_(C,N).

### Crystal data

**Table:**

[Cu(C_6_H_5_N_6_)_2_]	*F*(000) = 390
*M**_r_* = 385.86	*D*_x_ = 1.829 Mg m^−^^3^
Monoclinic, *P*2_1_/*c*	Melting point: 723 K
Hall symbol: -P 2ybc	Mo *K*α radiation, λ = 0.71073 Å
*a* = 6.6492 (6) Å	Cell parameters from 2942 reflections
*b* = 7.9093 (7) Å	θ = 3.0–27.5°
*c* = 13.5581 (12) Å	µ = 1.59 mm^−^^1^
β = 100.692 (2)°	*T* = 294 K
*V* = 700.65 (11) Å^3^	Rectangle, green
*Z* = 2	0.15 × 0.13 × 0.09 mm

### Data collection

**Table:**

Rigaku Mercury2 diffractometer	1363 independent reflections
Radiation source: fine-focus sealed tube	1228 reflections with *I* > 2σ(*I*)
graphite	*R*_int_ = 0.020
Detector resolution: 13.6612 pixels mm^-1^	θ_max_ = 26.0°, θ_min_ = 3.0°
ω scans	*h* = −8→7
Absorption correction: multi-scan (*CrystalClear*; Rigaku, 2005)	*k* = −8→9
*T*_min_ = 0.797, *T*_max_ = 0.870	*l* = −15→16
3743 measured reflections	

### Refinement

**Table:**

Refinement on *F*^2^	Primary atom site location: structure-invariant direct methods
Least-squares matrix: full	Secondary atom site location: difference Fourier map
*R*[*F*^2^ > 2σ(*F*^2^)] = 0.040	Hydrogen site location: inferred from neighbouring sites
*wR*(*F*^2^) = 0.109	H-atom parameters constrained
*S* = 1.07	*w* = 1/[σ^2^(*F*_o_^2^) + (0.0555*P*)^2^ + 1.0273*P*] where *P* = (*F*_o_^2^ + 2*F*_c_^2^)/3
1363 reflections	(Δ/σ)_max_ < 0.001
115 parameters	Δρ_max_ = 0.69 e Å^−^^3^
0 restraints	Δρ_min_ = −0.73 e Å^−^^3^

### Special details

**Table:**

Geometry. All e.s.d.'s (except the e.s.d. in the dihedral angle between two l.s. planes) are estimated using the full covariance matrix. The cell e.s.d.'s are taken into account individually in the estimation of e.s.d.'s in distances, angles and torsion angles; correlations between e.s.d.'s in cell parameters are only used when they are defined by crystal symmetry. An approximate (isotropic) treatment of cell e.s.d.'s is used for estimating e.s.d.'s involving l.s. planes.
Refinement. Refinement of *F*^2^ against ALL reflections. The weighted *R*-factor *wR* and goodness of fit *S* are based on *F*^2^, conventional *R*-factors *R* are based on *F*, with *F* set to zero for negative *F*^2^. The threshold expression of *F*^2^ > σ(*F*^2^) is used only for calculating *R*-factors(gt) *etc*. and is not relevant to the choice of reflections for refinement. *R*-factors based on *F*^2^ are statistically about twice as large as those based on *F*, and *R*- factors based on ALL data will be even larger.

### Fractional atomic coordinates and isotropic or equivalent isotropic displacement parameters (Å^2^)

**Table:**

	*x*	*y*	*z*	*U*_iso_*/*U*_eq_	
Cu1	0.5000	0.5000	0.5000	0.0265 (2)	
N6	1.0060 (4)	0.1967 (3)	0.61317 (18)	0.0302 (6)	
C6	0.7920 (5)	0.3986 (4)	0.3685 (2)	0.0260 (6)	
C5	0.9283 (4)	0.3088 (4)	0.4476 (2)	0.0263 (6)	
N4	0.6177 (4)	0.4748 (3)	0.37835 (19)	0.0294 (6)	
C4	0.8741 (5)	0.2899 (4)	0.5446 (2)	0.0258 (6)	
N3	0.8245 (4)	0.4167 (4)	0.27476 (19)	0.0345 (6)	
C3	1.1079 (5)	0.2369 (4)	0.4307 (2)	0.0322 (7)	
H3A	1.1439	0.2497	0.3680	0.039*	
N2	0.5399 (4)	0.5422 (4)	0.2875 (2)	0.0365 (6)	
C2	1.2364 (5)	0.1461 (4)	0.5041 (3)	0.0364 (7)	
H2A	1.3576	0.0997	0.4914	0.044*	
C1	1.1816 (5)	0.1263 (4)	0.5947 (2)	0.0349 (7)	
H1A	1.2646	0.0642	0.6446	0.042*	
N1	0.6643 (5)	0.5072 (4)	0.2266 (2)	0.0385 (7)	
N5	0.7116 (4)	0.3534 (3)	0.56979 (17)	0.0291 (6)	
H5A	0.7585	0.4076	0.6279	0.035*	
H5B	0.6431	0.2629	0.5866	0.035*	

### Atomic displacement parameters (Å^2^)

**Table:**

	*U*^11^	*U*^22^	*U*^33^	*U*^12^	*U*^13^	*U*^23^
Cu1	0.0241 (3)	0.0352 (3)	0.0227 (3)	0.00433 (19)	0.0104 (2)	0.00138 (19)
N6	0.0281 (14)	0.0330 (14)	0.0300 (13)	0.0006 (10)	0.0068 (11)	0.0029 (10)
C6	0.0267 (15)	0.0296 (14)	0.0243 (13)	−0.0035 (11)	0.0112 (11)	−0.0035 (11)
C5	0.0250 (15)	0.0270 (14)	0.0292 (15)	−0.0012 (11)	0.0108 (12)	−0.0019 (11)
N4	0.0280 (14)	0.0395 (14)	0.0223 (12)	0.0041 (11)	0.0087 (10)	0.0012 (10)
C4	0.0239 (15)	0.0258 (14)	0.0286 (14)	−0.0029 (11)	0.0076 (12)	0.0000 (11)
N3	0.0342 (15)	0.0477 (16)	0.0248 (12)	0.0030 (12)	0.0137 (11)	−0.0019 (11)
C3	0.0325 (17)	0.0347 (16)	0.0328 (16)	0.0011 (13)	0.0151 (13)	−0.0031 (13)
N2	0.0353 (16)	0.0513 (16)	0.0245 (13)	0.0073 (13)	0.0100 (11)	0.0046 (12)
C2	0.0280 (16)	0.0374 (17)	0.0464 (18)	0.0065 (13)	0.0141 (14)	−0.0028 (14)
C1	0.0297 (17)	0.0334 (16)	0.0393 (17)	0.0024 (12)	0.0002 (13)	0.0015 (13)
N1	0.0365 (17)	0.0561 (19)	0.0242 (13)	0.0054 (12)	0.0092 (12)	0.0040 (11)
N5	0.0277 (14)	0.0401 (15)	0.0229 (11)	0.0069 (11)	0.0135 (10)	0.0056 (10)

### Geometric parameters (Å, °)

**Table:**

Cu1—N5^i^	1.930 (2)	N4—N2	1.354 (4)
Cu1—N5	1.930 (2)	C4—N5	1.293 (4)
Cu1—N4	1.963 (2)	N3—N1	1.348 (4)
Cu1—N4^i^	1.963 (2)	C3—C2	1.386 (5)
N6—C1	1.358 (4)	C3—H3A	0.9300
N6—C4	1.369 (4)	N2—N1	1.302 (4)
C6—N4	1.335 (4)	C2—C1	1.354 (5)
C6—N3	1.336 (4)	C2—H2A	0.9300
C6—C5	1.455 (4)	C1—H1A	0.9300
C5—C3	1.380 (4)	N5—H5A	0.9000
C5—C4	1.435 (4)	N5—H5B	0.9000
			
N5^i^—Cu1—N5	180	C6—N3—N1	105.3 (2)
N5^i^—Cu1—N4	90.97 (10)	C5—C3—C2	122.1 (3)
N5—Cu1—N4	89.03 (10)	C5—C3—H3A	118.9
N5^i^—Cu1—N4^i^	89.03 (10)	C2—C3—H3A	118.9
N5—Cu1—N4^i^	90.97 (10)	N1—N2—N4	108.2 (3)
N4—Cu1—N4^i^	180	C1—C2—C3	118.5 (3)
C1—N6—C4	124.1 (3)	C1—C2—H2A	120.7
N4—C6—N3	110.1 (3)	C3—C2—H2A	120.7
N4—C6—C5	125.5 (3)	C2—C1—N6	120.3 (3)
N3—C6—C5	124.4 (3)	C2—C1—H1A	119.8
C3—C5—C4	118.8 (3)	N6—C1—H1A	119.8
C3—C5—C6	121.3 (3)	N2—N1—N3	110.1 (3)
C4—C5—C6	119.9 (3)	C4—N5—Cu1	132.2 (2)
C6—N4—N2	106.2 (2)	C4—N5—H5A	104.2
C6—N4—Cu1	128.3 (2)	Cu1—N5—H5A	104.2
N2—N4—Cu1	125.5 (2)	C4—N5—H5B	104.2
N5—C4—N6	119.4 (3)	Cu1—N5—H5B	104.2
N5—C4—C5	124.5 (3)	H5A—N5—H5B	105.5
N6—C4—C5	116.1 (3)		
			
N4—C6—C5—C3	−178.6 (3)	C6—C5—C4—N6	176.9 (3)
N3—C6—C5—C3	1.2 (5)	N4—C6—N3—N1	0.1 (3)
N4—C6—C5—C4	3.1 (4)	C5—C6—N3—N1	−179.8 (3)
N3—C6—C5—C4	−177.1 (3)	C4—C5—C3—C2	0.5 (5)
N3—C6—N4—N2	0.1 (4)	C6—C5—C3—C2	−177.8 (3)
C5—C6—N4—N2	179.9 (3)	C6—N4—N2—N1	−0.2 (4)
N3—C6—N4—Cu1	−176.5 (2)	Cu1—N4—N2—N1	176.5 (2)
C5—C6—N4—Cu1	3.4 (4)	C5—C3—C2—C1	0.8 (5)
N5—Cu1—N4—C6	−7.2 (3)	C3—C2—C1—N6	−1.1 (5)
N5—Cu1—N4—N2	176.9 (3)	C4—N6—C1—C2	0.1 (5)
C1—N6—C4—N5	−179.3 (3)	N4—N2—N1—N3	0.2 (4)
C1—N6—C4—C5	1.2 (4)	C6—N3—N1—N2	−0.2 (4)
C3—C5—C4—N5	179.0 (3)	N6—C4—N5—Cu1	175.8 (2)
C6—C5—C4—N5	−2.6 (4)	C5—C4—N5—Cu1	−4.7 (5)
C3—C5—C4—N6	−1.4 (4)	N4—Cu1—N5—C4	8.1 (3)

Symmetry codes: (i) −*x*+1, −*y*+1, −*z*+1.

### Hydrogen-bond geometry (Å, °)

**Table:**

*D*—H···*A*	*D*—H	H···*A*	*D*···*A*	*D*—H···*A*
N5—H5A···N2^i^	0.90	2.50	2.902 (4)	108

Symmetry codes: (i) −*x*+1, −*y*+1, −*z*+1.
